# Quorum Sensing, Biofilm, and Intestinal Mucosal Barrier: Involvement the Role of Probiotic

**DOI:** 10.3389/fcimb.2020.538077

**Published:** 2020-09-25

**Authors:** Zhaoxi Deng, Xin M. Luo, Jianxin Liu, Haifeng Wang

**Affiliations:** ^1^Ministry of Education Key Laboratory of Molecular Animal Nutrition, College of Animal Science, Zhejiang University, Hangzhou, China; ^2^Department of Biomedical Sciences and Pathobiology, Virginia Polytechnic Institute and State University, Blacksburg, VA, United States

**Keywords:** quorum sensing, biofilm, bacteria, intestine, mucosal barrier, probiotic

## Abstract

The intestine is a particularly dynamic environment in which the host constantly interacts with trillions of symbiotic bacteria called the microbiota. Using quorum sensing (QS) communication, bacteria can coordinate their social behavior and influence host cell activities in a non-invasive manner. Nowadays, a large amount of research has greatly spurred the understanding of how bacterial QS communication regulates bacterial cooperative behaviors due to coexistence and host-microbe interactions. In this review, we discuss bacterial QS in the gut and its role in biofilm formation. As a biological barrier, the mucosal immune system can effectively prevent pathogenic microorganisms and other immunogenic components from entering the internal environment of the host. We focus on the relationship between biofilm and intestinal mucosal immunity, and how probiotic bacteria may regulate them. This review is to provide a theoretical basis for the development of new techniques including probiotics targeting the intestinal barrier function, thereby improving gut health.

## Introduction

In the intestinal micro-ecological environment, there are 100 trillion microbes, including bacteria, fungi, viruses, protozoa. Ninety-nine percent of them are bacteria. The stable and diverse intestinal microbiota ecosystem has complex digestive and metabolic functions (Lan and Jianqiong, 2017). Intestinal homeostasis is dependent on the cooperation of immune cells residing on the intestinal epithelium and the gut microbiota (Burger et al., 2018). Probiotics have been described as “live microorganisms which, when administered in adequate amounts, confer a health benefit on the host” (O'Toole and Cooney, 2008; Kang and Sinhyeog, 2015). Biofilms, as multicellular aggregates on living substrates or inanimate, is a way of life for many microbes to proliferate. It is worth noting that quorum sensing (QS) plays a crucial role in biofilm formation. The occurrence of probiotic biofilms in the healthy gut could extend bacterial residence time and promote the exchange of nutrients between the host and the microbiota (Hooper and Gordon, 2001). Probiotic bacteria can colonize permanently in the host mucosa through formation of biofilms, which subsequently prevent the colonization of pathogens (Halfvarson et al., 2017). The mucosal immune system acts as a biological barrier, preventing pathogenic microorganisms, and other immunogenic components from passing through the mucosa into the host. Along with the intestinal epithelial barrier, this biological barrier formed by intestinal immune cells and their secretion provides protection against infectious threats. At the same time, the mucosal immune system supports an ecosystem with multiple metabolic and immune functions that are critical to the survival of the host (Lan and Jianqiong, 2017). Therefore, the functions of these three components, probiotics, biofilms, and mucosal immunity, can work in tandem to maintain intestinal immune homeostasis.

## Bacterial QS in the Intestine

**Figure 1:**
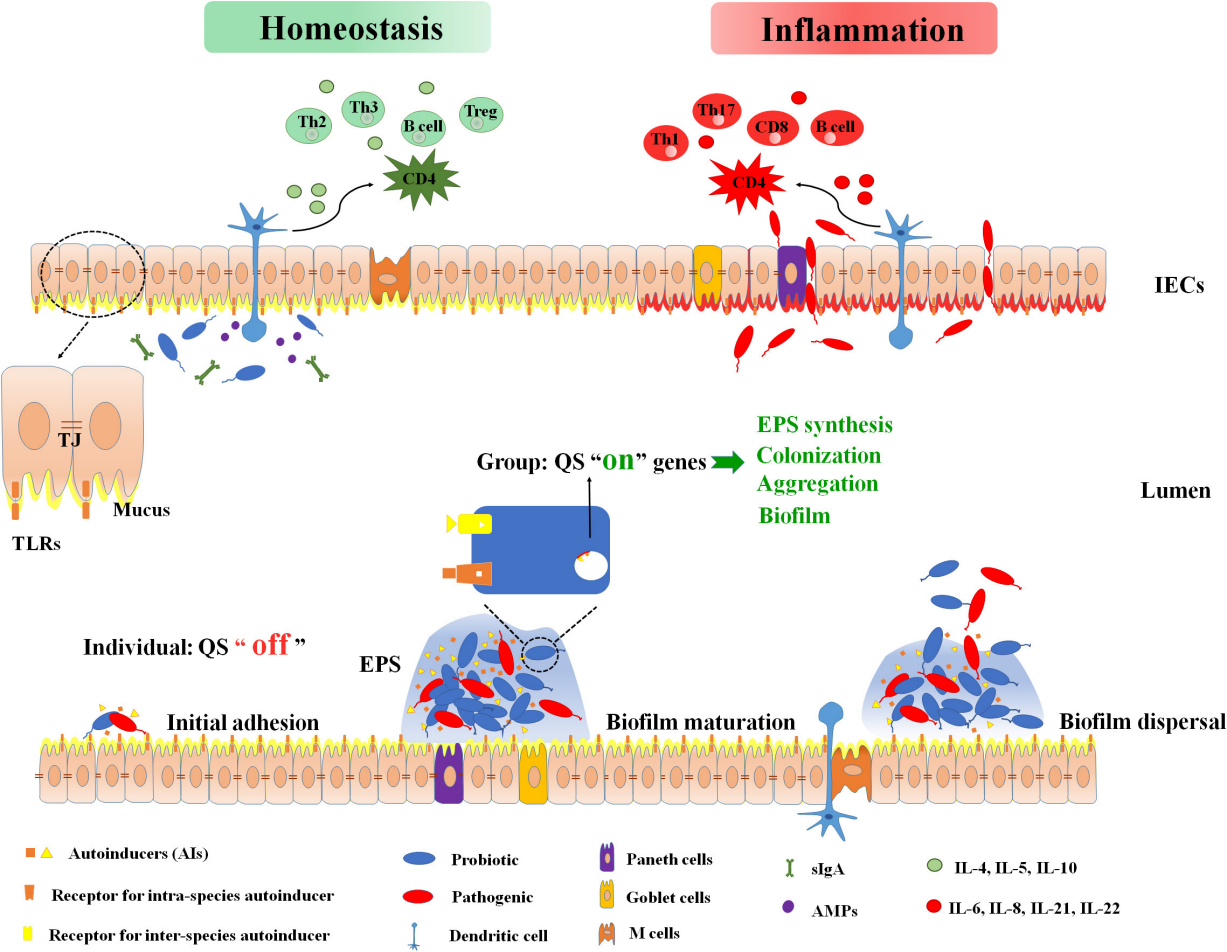
Intestinal immune barrier associated with biofilm and microbiota. Bacteria sense the density of themselves or surrounding bacteria, and secrete autoinducers (AIs) to the extracellular. When signal molecules reach at a certain threshold, the quorum sensing turns from “off” to “on”. It is recognized by the bacterial specific receptor, and finally the target gene expression is activated. These genes control bacterial extracellular polysaccharides (EPS) synthesis, aggregation, colonization, biofilm formation, and so on. At homeostasis status, mucus, antimicrobial peptides (AMPs), and intestinal B-cell secretory immunoglobulin A (sIgA) isolate intestinal bacteria from the intestinal lumen. Anti-inflammatory cytokines (IL-4, IL-5, IL-10) protect the intestinal mucosa epithelium and fix the symbiotic flora on the protective mucus. Dendritic cells (DCs) are able to produce a tolerant response by down-regulating the nuclear factor kappa-B (NF-κB) signaling cascade when DCs activation is induced by symbiotic bacteria. This establishes a symbiotic relationship between the organism and the symbiotic flora. At inflammation status, loss of intestinal barrier function causes translocation of bacteria across intestinal epithelial cells (IECs). The pathogen activates toll-like receptors (TLR) and subsequently induces the release of inflammatory cytokines IL-6, IL-8, IL-21, and IL-22. These cytokines will further up-regulate the NF-kB system and stimulate CD4 to differentiate into inflammatory Th1, Th17, and CD8-cytotoxic subpopulations. AIs, autoinducers; AMPs, antimicrobial peptides; DCs, dendritic cells; EPS, extracellular polysaccharides; IECs, intestinal epithelial cells; QS, quorum sensing; sIgA, secretory immunoglobulin A; TJ, tight junction; TLR, toll-like receptors.

The microbiota colonizing the gastrointestinal tract regulates the immune function of the gastrointestinal tract and mucous membranes, which is important for the health of the host. Despite great challenges in the gut, such as stomach acid and intestinal bile, the microbiota has proven to have an exceedingly stable structure (Thompson et al., 2016). The stability of the intestinal microbiota is dependent on QS (Thompson et al., 2016). QS is a cell-to-cell communication process dependent on extracellular signaling molecules secreted by bacteria, called autoinducers (AIs). All bacterial QS systems consist of signaling molecules, sensing molecules, and downstream regulatory proteins. When the number of bacteria reaches a certain threshold, AIs bind to the corresponding QS receptors on the bacterial surface at a high density. The receptors upon internalization bind to the corresponding binding domains of genes to regulate physiological functions that individual cells cannot perform independently (Melissa and Bonnie, 2001), leading to an autoinduction feedback loop that promotes the synchronized development of bacterial populations (Rutherford and Bassler, 2012). QS can result in a cooperative change in bacterial gene expression (e.g., expression of virulence factors) and bacterial behaviors (e.g., biofilm formation) (Whiteley et al., 2017) (Figure 1).

QS is present in both Gram-positive and Gram-negative bacteria. The types of AIs in bacterial QS include auto-inducing peptides (AIPs), acylated homoserine lactone (AHL), pseudomonas quinolone signal (PQS), autoinducers-2 (AI-2), autoinducers-3 (AI-3), among others. *Escherichia coli* in the gut is regulated by at least three QS signaling molecules. One of the signaling molecules is AHL, which is mainly found in Gram-negative bacteria. Another is AI-2, which consists of 4,5-dihydroxy-2,3-pentanedione (Pereira et al., 2013). The last one is AI-3, which produces a cascade of amplification reactions similar to epinephrine, suggesting that AI-3 may be structurally similar to adrenergic/norepinephrine. QS systems *AinS/AinR, LuxI/LuxR*, and *LuxS/LuxPQ* found in *Vibrio cholerae* regulate bacterial colonization and subsequent biofilm formation (Jung et al., 2015). The ease of accessibility and non-invasive sampling have made oral biofilms a model for human biofilms (Tytgat et al., 2019). In contrast, the situation in the gut is less straightforward, whether QS can be used to strengthen the viability of beneficial bacteria in the gut is an underexplored topic.

## Biofilm

Bacteria can exist in nature in the form of free-floating planktonic bacteria or sessile colonies of microbes forming biofilms (Probert and Gibson, 2002). Bacterial survival in the gut, either temporarily or permanently, is dependent on their ability to colonize. Temporary bacteria are microbes that enter the gut from the external environment during adulthood and do not colonize permanently. Conversely, permanent bacteria establish a long-term relationship with the host as permanent members of the microbiota (Ivanov and Honda, 2012). Biofilm is an organized microbial aggregate that live within a matrix of extracellular polysaccharides (EPS), irreversibly adhered to a substratum or interface or to each other (Costerton et al., 1995). The transition of bacteria from a free-living state to a polycellular population is a complicated and dynamic process that undergoes multiple changes, such as cellular reprogramming, variations in expression of cell surface molecules, and the generation of virulence factors (Kostakioti et al., 2013). The concentration of QS signaling molecules in biofilms can be 1,000 times higher than that in environments where planktonic bacteria inhabit (Flemming et al., 2016). Studies have also shown that the thickness, biomass, activity, and morphology of biofilm depend on the availability of nutrients in the environment. Microorganisms grown under sufficient nutrition produced higher sessile biomass and thicker biofilm (Salgar-Chaparro et al., 2020). Microorganisms located on the surface of the biofilm are more active because they are more likely to obtain nutrients and release metabolic waste. However, due to the diffusion barrier created by EPS and other biofilm components, microorganisms at the bottom area of the biofilm are exposed to more nutrient depletion conditions (Anwar et al., 1992). The development of culture-independent, high-throughput molecular techniques, and isolate-independent, non-target metabolomics technology have enabled the identification of previously unknown bacterial species and unidentified metabolite, thereby providing novel insights into the compositional diversity, functional capacity, and metabolic of biofilm microbiota, and further elaborating the relationship between the biofilm formation and the organism metabolism.

### EPS as a Biofilm Stabilizing Component

Biofilms are generally composed of microbial cells and secreted EPS, which account for 90% of the total biomass. EPS covers ~45% of the surface of Gram-negative bacteria, and extends 30 nm or more to the surrounding media (Hwang et al., 2012). The cells are encapsulated in a self-made matrix box that consists of sugar polymers as main components along with polymers of proteins that facilitate the viscoelastic property of the biofilm (Limoli et al., 2015; Gupta et al., 2016).

The encapsulation of EPS in the biofilm provides many important advantages for the cells. EPS is polyanionic and absorbs nutrients from the surrounding fluid to promote cell growth (Singh et al., 2017). EPS is essential for maintaining the stability of the biofilm structure. For example, the *icaADBC* gene in *Staphylococcus aureus* encodes an EPS called polysaccharide intercellular adhesion (PIA). Confocal microscopic imaging showed that a weak PIA-producing strain of *S. aureus* forms a biofilm of simple morphology, while a strong PIA-producing strain forms small, tight, mushroom-like colonies separated by wide channels (Lister and Horswill, 2014). EPS in?uences the biochemical characteristics of the cell such as hydrophobicity and surface charge and alters the adhesive properties of the biofilm. The carboxylate and amine functional groups contained in the EPS facilitate bacterial adhesion to the intestinal surface (Peterson et al., 2015). These EPS-mediated changes support cellular recognition and further stimulate adhesion and aggregation (Ding et al., 2015; Peterson et al., 2015). Finally, cells encapsulated in EPS have a better ability to withstand external environmental stress (Pang et al., 2005), such as antibiotics and attack from immune cells (Goldberg, 2002; Mulcahy et al., 2014). In bacterial biofilms with high concentrations of bacteria, intercellular signaling, or QS is likely to occur within the EPS matrix. For example, *Pseudomonas aeruginosa* has two sets of QS systems, las (LasI/LasR) and rhl (RhlI/RhlR). The las system produces glucose-rich EPS matrix that regulates biofilm formation (Davies et al., 1998). Studies on *E. coli* have also shown that AI-2 can regulate biofilm formation by affecting the production of EPS (Kim et al., 2010). Together these evidences suggest that bacterial secretion of EPS, which is regulated by QS, plays a critical role in biofilm formation. However, it is not clear which QS signal has greater importance or whether QS signals work together during biofilm formation.

### Role of QS in Biofilm Formation

The process of biofilm formation is divided into the following four stages: initial attachment to the surface, microcolony formation, biofilm maturation, and differentiation, and detachment of the biofilm (Stoodley et al., 2002). Once the microbes attach to the surface, bacterial cell proliferation, and intercellular adhesion would occur. PIA facilitates intercellular adhesion and biofilm accumulation in *Staphylococci* (Beauvais et al., 2007), whereas poly-β-1,6-N-acetylglucosamine is a polysaccharide involved in intercellular adhesion and surface attachment in the formation of *E.coli* biofilms (Anantha et al., 2000). The microbes then develop into microcolonies and are surrounded by EPS. Intercellular signaling or QS takes place in the EPS matrix at this point. By manipulating EPS-based phenotypes and the QS system, one could promote or inhibit biofilm formation. During the process of detachment, bacterial flora within the biofilm releases different saccharolytic enzymes. For example, *P. aeruginosa* produces alginate lyase, *Streptococcus equi* produces hyaluronidase, and *E. coli* produces N-acetyl-heparosan lyase. These enzymes help the colony to release and attach to a new area (Otto, 2013; Jamal et al., 2018). There are different QS systems affecting the formation of different bacterial biofilms. The *comCDE* gene in *Streptococcus mutans* encodes a peptide signal molecule named competence-stimulating peptide. Using mutants lacking *comC, comD*, and *comE*, it was found that although all mutants formed biofilms, the mutant biofilms lacked the structural integrity found in wild-type biofilms (Li et al., 2002). Some microbes contain Ser/Thr kinase genes, which control the biosynthesis of signaling molecules to regulate biofilm formation. For example, the formation of biofilms for *Bacillus subtilis* and *S. aureus* is regulated by a Ser/Thr kinase. This study also found that *PrkC* from *B. subtilis* is a Ser/Thr kinase similar to a eukaryotic sensor, and the mutation of *PrkC* leads to a decline in biofilm growth, indicating that this protein plays an important role in biofilm formation (Madec et al., 2010). *Stk1* is a *PrkC* homolog in *S. aureus*, which inactivates the *luxS* protein by phosphorylation and eliminates the production of AI-2, thereby affecting biofilm formation (Cluzel et al., 2010). Taken together, the regulation of the biofilm-forming process is rather complicated and includes multiple elements.

### Methods for Biofilm Detection

At present, there are many qualitative and quantitative detection methods for biofilms. Static methods are especially meaningful for examining early events in biofilm development, such as crystal violet staining, scanning electron microscope (SEM), confocal laser scanning microscope (CLSM), calgary biofilm equipment, and biofilm ring test (BRT). However, these closed models do not allow substances to flow in or out, leading to nutrient consumption and accumulation of metabolites, which gradually changed the experimental conditions (Roy et al., 2018). Dynamic methods, such as robbins reactors, rotating or rotating disk reactors, and trickle flow reactors, can precisely control the nutrient delivery and flow, which will better simulate the internal environment (Merritt et al., 2005). However, these dynamic methods require specialized equipment and are confronted with technical challenges. Therefore, each method has advantages and disadvantages, which can be comprehensively considered according to the purpose, demand, and cost of the experiment.

## Intestinal Mucosal Barrier and Microbial Biofilm

The intestinal mucosa is the interface between the outside and the internal environment, and one of the most important obstacles to prevent entry from the external environment. The intestinal mucosa is made of an epithelial cell lining that includes goblet cells, M cells, Paneth cells, enteroendocrine cells and absorptive intestinal cells (Ott et al., 2004; Peterson and Artis, 2014). The intestinal epithelium consists of a single layer of intestinal epithelial cells (IECs) sealed with tight junctions (TJs) to physically separate bacteria from the sterile tissue (Turner, 2009). The intestinal epithelium experiences quick and permanent self-renewal, which improves cell-cell functional integrity and intestinal barrier function. The diverse functions of intestinal epithelium include barrier function, nutrient absorption, water retention, and maintenance of immune homeostasis (Figure 1). A complete barrier includes a physical defense mechanism related to the mucosal surface, IECs, and cells associated with innate and acquired immune systems (Garrett et al., 2010).

### Microbial Biofilm as a Physical Barrier

One of the niches that have been extensively studied about microbial biofilms is the intestinal tract of an organism, which is also considered to be the largest immune organ in our body. The main physiological functions of the intestine are to absorb nutrients, digest food, and eliminate unnecessary waste. The gut is also the most invasive site for many bacterial and viral pathogens (Palmer et al., 2007). Despite direct exposure to a large number of microorganisms and foreign antigens, a unique intestinal mucosal immune system maintains the homeostasis of the gut. The close contact of the bacterial consortium with the host is also related to the formation of biofilms, which promote synergy between bacteria and the host and enhance host defense capabilities. Conversely, the intestinal immune network supports the growth of specific commensal bacteria (Dishaw et al., 2016). There is constant interaction between the epithelial cells and the gut microbiota, both of which have been implicated in the regulation of intestinal barrier function (Natividad and Verdu, 2013). The commensal microbiota is able to shape the intestinal barrier structure by controlling physiological paracellular permeability and enhancing the mucus layer (Hayes et al., 2018).

The intestinal barriers, either physical or biological, prevent pathogens from entering the body. They consist of four interdependent components, namely the continuous intestinal epithelium, the mucus layer, the mucosal immunity and the intestinal microbiota (Iacob et al., 2019). The epithelial surface of the intestinal tract constitutes a physical barrier against the “outside,” thereby providing a first layer of resistance to infections. The second layer of defense against invading pathogenic microorganisms and immunogenic components is the mucus layer, a hydrated gel covering the surface of the intestinal mucosa. The mucus layer consists of mucin secreted by goblet cells and antimicrobial proteins produced by Paneth cells. It creates a protective environment for the gut microbiota and especially for bacteria that thrive near to the epithelial cells (Cornick et al., 2015). The third layer of defense is provided by the intestinal mucosal immunity, including gut-associated lymphoid tissues (GALT), secretory immunoglobulin A (sIgA), antimicrobial peptides (defensins or lysozymes) and mucosal immune cells (such as Th1, Th2, and Treg cells) (Goto et al., 2016; Kurashima and Kiyono, 2017). The fourth layer of defense is the intestinal microbiota. The microbiota prevents pathogens from invading the intestinal mucosa through competition. This mechanism is called “colonization resistance,” which is associated with QS (Tytgat et al., 2019). Mucin, together with sIgA, motivates microbial activity by binding to a “normal, healthy” microbiota to promote biofilm formation (Everett et al., 2004).

Biofilms provide a protective shell for pathogenic bacteria to evade host defense (Tytgat et al., 2019). They are an ideal environment for pathogenic bacteria to build virulence, so the occurrence of some mature biofilms on healthy tissues may be an early warning signal for the transition to a damaged gut. For example, an increased amount of adherent invasive *E. coli* forming biofilms is associated with the occurrence of ulcerative colitis (Halfvarson et al., 2017; Shawki and McCole, 2017). On the other hand, biofilms formed in the healthy gut would exert a beneficial function on the host by boosting the functions served by the microbiota, such as enhancing host defense (Sonnenburg et al., 2004). These biofilms greatly prolong the residence time of bacteria in the gut, thereby enhancing the exchange of nutrients between microbiota and the host (Hooper and Gordon, 2001). The formation of probiotic biofilms is a favorable feature for the host, promoting the colonization and longer persistence of beneficial bacteria in the gut mucosa, thereby precluding colonization of pathogens (Terraf et al., 2012) (Figure 1).

### Intestinal Immune Barrier Associated With Biofilm and Microbiota

The strategic position of IECs between intestinal microbes and mucosal immune cells determines its significant role in signal transduction (Jeremy et al., 2013). Signals from intestinal bacteria may promote the integrity of the epithelial barrier by adjusting intestinal permeability while controlling the rate of IEC proliferation (Ivanov and Littman, 2011). Epithelium develops an intimate network system through tight junction proteins (zonulins, occludins, and claudins) that are tightly controlled by a variety of signals (Marchiando et al., 2010). IECs also have fundamental immuno-regulatory functions. They use pattern-recognition receptors (PRRs) as sensors and then convey the corresponding signals to immune cells (Peterson and Artis, 2014). In a steady-state environment, the microbiota, which is readily recognizable by PRRs, influences the development and function of diverse immune cells, such as plasma cells, Th17 cells, Treg cells, NK cells, and dendritic cells (Honda and Littman, 2011).

Goblet cells are the most plentiful secretory epithelial cells in the gut, which has a primary function to secrete highly glycosylated proteins named mucin to the intestinal lumen. Mucin can self-assemble into a protective mucus layer, coating the surface of epithelial cells (Pelaseyed et al., 2014; Peterson and Artis, 2014). Bacterial colonization induces the intestinal goblet cells to secrete mucin. *Bacteroides thetaiotaomicron* is a commensal bacterium found in the human gut, which regulates goblet cell di?erentiation and mucin-related gene expression by upregulating *KLF4*. *Faecalibacterium prausnitzii* counteracts the influence of *B. thetaiotaomicron* to avoid excessive mucus production, which is necessary for healthy epithelial structure (Wrzosek et al., 2013). Moreover, mucin promotes bacterial clearance, regulates biofilm formation and virulence factors, while ensuring the survival of commensals that would in turn fight against pathogens (Sicard et al., 2017). The O-glycan structure of the mucus layer is a receptor for bacterial adhesion and allows the colonization of healthy probiotics such as *Lactobacillus* and *Bifidobacterium* (Bergstrom and Xia, 2013). Although the defense offered by mucins is generally considered passive and easily destroyed by pathogenic microbes, recent studies have begun to challenge the concept that goblet cells are ineffective in sensing and responding to infections (Pelaseyed et al., 2014).

The uneven distribution of Paneth cells along the intestinal tract may form a gradient of bacterial diversity and density from the proximal to distal regions of the small intestine (Denise et al., 2005). The production of AMPs by Paneth cells is dominant in maintaining intestinal homeostasis, and AMPs are abundantly present in the mucus layer. AMPs also regulate the diversity and density of the gut microbiota, thereby protecting the intestinal epithelium from foreign pathogens (Wang et al., 2018). The secretion capability of Paneth cells is one of the intestinal barrier functions that promotes intestinal homeostasis. For example, *B. thetaiotaomicron* induces the secretion of angiogenin in Paneth cells, angiogenin acts as a new class of antimicrobial peptide with the ability to selectively protect intestinal crypts from pathogenic bacteria (Hooper et al., 2003).

Commensal bacteria induce sIgA production and its export to the intestinal lumen (Macpherson et al., 2000). sIgA is an antibody class produced by plasma cells in the lamina propria and is a distinctive immunological characteristic of the intestinal mucosal immune system (Tokuhara et al., 2010). sIgA promotes barrier protection against enteric pathogens by binding to surface molecules expressed by pathogens and by neutralizing their toxins. It is worth mentioning that sIgA is known for its ability to inhibit biofilm formation by agglutinating immune rejection, preventing translocation of the epithelial barrier. The release of sIgA relies on colonization of bacteria in the gut. There is a significant decrease in sIgA in the intestinal tract of germ–free mice (Macpherson et al., 2000). Indeed, sIgA coats 24–74% of the microbiota (Nicolas et al., 2012). sIgA is able to recognize bacteria through different modes, and is thus divided into either cross–reactive IgA or specific IgA (Brandtzaeg, 2013). sIgA prevents bacterial aggregation and adhesion by keeping cells in a planktonic state and down-regulating the expression of biofilm-associated genes in pathogens (Kavanaugh et al., 2014). A recent study also pointed out that sIgA, together with mucin, can play a role in microbial activity by binding members of the “normal, healthy” microbiota to support biofilm formation (Everett et al., 2004) (Figure 1). At present, most studies on gut biofilms are still *in vitro*, which cannot truly reflect the effect of biofilm formation on the intestinal barrier *in vivo*. The increase in organoid research has provided new ideas for the research of intestinal biofilm. Organoid is a cultured 3D cell structure, which is similar to the intact intestinal structure and function *in vivo*, and can effectively simulate organ function, composition, and development characteristics. Intestinal organoid culture technology has been applied to inflammatory bowel disease (IBD), intestinal malignancies, and the interaction of specific intestinal flora with the host (Sato et al., 2009; Schwitalla et al., 2013; Dedhia et al., 2016). However, organoid culture lacks the participation of intestinal microbiota, vascular endothelial cells and immune cells, so it can only reflect the physiological or pathological characteristics of local tissues, ignoring the role of other factors in this process (Dedhia et al., 2016).

### Intestinal Biofilm and Barrier Related With Bacterial SCFA

Short-chain fatty acids (SCFA) are one of the important metabolites of gut microbes, mainly composed of acetate, propionate, and butyrate, but others, such as lactate and valerate, are also produced by microbiota (LeBlanc et al., 2017). As signal molecules, they have various effects on the host, such as regulating host metabolism, maintaining intestinal homeostasis, and strengthening the immune system (Ashida et al., 2011). Healthy gut microbiota produces an adequate and balanced SCFA as an important way to prevent pathogen infection (Round and Mazmanian, 2009). Acetic acid inhibits extracellular polysaccharides and shows anti-quorum sensing activity in *E. coli*, which ultimately leads to a reduction in biofilm formation (Amrutha et al., 2017). On the other hand, acetic acid can also promote the biofilm formation of *Bacillus subtiliss* to play a probiotic role (Chen et al., 2015). However, the mechanism of how these molecules affect biofilm is currently unknown. SCFA may regulate the expression of virulence genes by affecting quorum sensing. SCFA significantly up-regulated the expression of luxS encoding Autoinducer-2 (AI-2) in *Salmonella typhimurium* T7, thus it is speculated that SCFA may inhibit quorum sensing by blocking AI-2 rather than down-regulating genes involved in the production of signaling molecules (Banerjee and Ray, 2016). Butyrate has been shown to alleviate the abnormal expression of ZO-1 and reduce liposaccharides translocation, thereby inhibiting macrophage activation and the production of pro-inflammatory cytokine (Liu et al., 2014). The data also indicated that butyrate can inhibit the activity of TNF-α, IL-6, and myeloperoxidase to alleviate inflammation by preventing NF-κB (Qiao et al., 2014). In addition, it has been confirmed that macrophages differentiated in the presence of butyrate can increase antibacterial activity even without an increased inflammatory cytokine response (Schulthess et al., 2019). Whether and how short-chain fatty acids can regulate the intestinal barrier function by affecting the expression of quorum sensing-related genes and gut microbiome deserves to be elucidated.

## Formation of Probiotic vs. Pathogenic Biofilms and Their Effects on Intestinal Mucosal Immunity

Ideally, the human lives in harmony with its gut microbiota in a state that promotes physiological resilience, however, dysbiosis can result from challenge such as infections, lifestyle, and unbalance nutrition (Dethlefsen and Relman, 2011; Sanders et al., 2019). Intestinal diseases are often associated with severe imbalance of microbiota. IBD and colorectal cancer (CRC) linked to a disruption of the healthy microbiota and mucosal epithelium severely affects gut-related biofilm (Tytgat et al., 2019). The outgrowth of thick polymicrobial pathogenic mucosal biofilms marks the transition between a healthy and diseased microbiota. The healthy ecological state of the microbiota, that is, commensal coexistence in microcolonies with the host, can be disrupted by environmental factors and pathogens supporting the outgrowth and transformation of healthy microbial consortia to pathogenic mature biofilms (Tytgat et al., 2019). The average density of the colony biofilm in IBD was found to be a 100-fold higher than in healthy individual (Swidsinski et al., 2005). *Fusobacterium nucleatum* causes intestinal diseases in the form of invasive biofilms. At the same time, mature biofilms have also appeared in adjacent healthy tissues infected with CRC and IBD. It is possible that biofilm is an early warning signal for intestinal diseases. To some extent, biofilms provide a protective environment that promotes the escape of host defense mechanisms, and further aggravate the diseases (Hoarau et al., 2016). Although antibiotics can remove the biofilm of most harmful bacteria, biofilms can regenerate rapidly during chronic wound healing, indicating the presence of persistent cells in the biofilm.

Conversely, for probiotics, we want to prolong their residence time in the gut and maximize their probiotic effects. Biofilm formation depend on adhesion, self-aggregation and co-aggregation as significant features of bacteria (Chervinets et al., 2018). The most desirable characteristic of probiotic strains is their good adhesion which prolongs their stay in the gut, which effectively enhance barrier function, increase IEC activity and stimulate protective responses of IECs to maintain intestinal epithelial homeostasis (Sazawal et al., 2006; Chervinets et al., 2013) (Figure 1). Probiotics can prevent pathogen colonization and mucosal infection by combating nutrients and releasing small-molecular-weight antibiotics. On the other hand, it protects or treats intestinal diseases by stabilizing the intestinal mucosa, increasing mucus secretion, and improving bowel movements (Delgado et al., 2010). It has been found that if the adhesion of the pathogen to the mucosa is inhibited *in vitro* and biofilm formation is diminished, which reduces the activity of *Helicobacter pylori* (Chenoll et al., 2011). *E. coli Nissle* 1917 has good biofilm formation ability, which is stronger than that of *Enteropathogenic Escherichia coli* (EPEC) and *Enterotoxigenic Escherichia coli* (ETEC), and competes with these strains during biofilm formation. Therefore, the *E. coli Nissle* 1917 can be used as a probiotic against various intestinal diseases (Hancock et al., 2010). *Lactobacillus plantarum* and *ETEC* recognize the same adherence sites on the intestinal epithelial surface, the pathogenicity of *ETEC* to the human body can be attenuated by promoting the adhesion of *L. plantarum*.

The importance of probiotics in maintaining human health is unquestionable, and research on healthy biofilm has recently become a hot topic. A restorative or protective effect of certain strains of probiotics on the fecal microbial community and host physiology, e.g., alleviation of gastrointestinal symptoms where dysbiosis is present or where the microbiota is perturbed (McFarland, 2014). Probiotics are deemed to be a dietary approach to the modification of the gut microbiota to improve host health (Gibson et al., 2017). Various tests have indicated that a wide range of applications of probiotic are associated with the benefits of preventing infection and disease (Martinez et al., 2015; Forsberg et al., 2016). It is effective for the treatment of acute infectious diarrhea, antibiotic-associated diarrhea, irritable bowel syndrome and functional gastrointestinal diseases (Wilkins and Sequoia, 2017). The intestinal microbiota has been used as a new therapeutic target to decrease the chronic inflammation (Reena et al., 2013). Therefore, probiotics are expected to enhance and improve the resident intestinal microbiome (Klatt et al., 2013; Amara and Shibl, 2015). In the future, modulation targeting the microbiota will probably be a powerful weapon against intestinal diseases as genomic and metabolomic approaches promise to uncover important links between the microbiota and intestinal health.

## Conclusions and Perspectives

In summary, the formation of bacterial biofilm is a complex system engineering, and QS system is one of the main regulatory systems, which is essential for intestinal homeostasis. In recent years, the bacterial QS system has become a research hotspot, but most of the research focuses on how the QS system controls the formation of biofilms of pathogenic bacteria. There is little research on how to regulate the biofilm formation of probiotics. Therefore, focusing on the molecular mechanism of QS system to regulate the formation of probiotic biofilms plays an important role in maximizing the benefits of probiotics in the intestine.

## Author Contributions

ZD and HW wrote the manuscript. XL and JL revised the manuscript. HW designed, revised, and finalized the manuscript. All authors contributed to the article and approved the submitted version.

## Conflict of Interest

The authors declare that the research was conducted in the absence of any commercial or financial relationships that could be construed as a potential conflict of interest.
